# Signet ring cell cancer of stomach and gastro-esophageal junction: molecular alterations, stage-stratified treatment approaches, and future challenges

**DOI:** 10.1007/s00423-021-02314-6

**Published:** 2021-09-10

**Authors:** Naveena A. N. Kumar, Anmi Jose, Nawaz Usman, Keshava Rajan, Murali Munisamy, Preethi S. Shetty, Mahadev Rao

**Affiliations:** 1 Department of Surgical Oncology, Manipal Comprehensive Cancer Care Center, Kasturba Medical College, Manipal Academy of Higher Education (MAHE), Manipal, Karnataka 576104 India; 2 Department of Pharmacy Practice, Manipal College of Pharmaceutical Sciences, Manipal Academy of Higher Education (MAHE), Manipal, Karnataka 576104 India

**Keywords:** Signet ring cell cancer, Stomach cancer, Gastro-esophageal junction cancer, Molecular alterations, E-cadherin, Stage-stratified treatment

## Abstract

**Purpose:**

There has been an increase in the incidence of signet ring cell cancer (SRCC) of the stomach and gastro-esophageal junction (GEJ). The multistage carcinogenesis involving genetic and epigenetic aberrations may have a major role in the increasing incidence of SRCC. Although there are numerous studies on the prognostic value of SRCC, they are markedly inconsistent in their results, making it impossible to draw any meaningful conclusions. We aimed to examine the available evidences on molecular alterations and stage-stratified treatment approaches in SRCC of the stomach and GEJ.

**Methods:**

A systematic search was carried out in PubMed. Studies available in English related to SRCC of stomach and gastro-esophageal junction were identified and evaluated.

**Results:**

This study reviewed the current evidence and provided an insight into the molecular alterations, stage-stratified treatment approaches, and future challenges in the management of SRCC of the stomach and GEJ. Specific therapeutic strategies and personalized multimodal treatment have been recommended based on the tumor characteristics of SRCC.

**Conclusion:**

Multistage carcinogenesis involving genetic and epigenetic aberrations in SRCC is interlinked with stage-dependent prognosis. Specific therapeutic strategy and personalized multimodal treatment should be followed based on the tumor characteristics of SRCC. Endoscopic resection, radical surgery, and perioperative chemotherapy should be offered in carefully selected patients based on stage and prognostic stratification. Future studies in genetic and molecular analysis, histopathological classification, and options of multimodality treatment will improve the prognosis and oncological outcomes in SRCC of gastric and GEJ.

## Introduction

Gastric cancer is the fifth most common cancer worldwide and third leading cause for cancer-related mortality [1]. Though the overall incidence is decreasing, there is a marked regional variation bound by environmental factors, with the incidence being high in Eastern Asia and low in Northern America and Europe [1]. The incidence of gastro-esophageal junction (GEJ) cancers, however, has been increasing in high income countries [1, 2]. Recent studies have found an increase in incidence of GEJ/gastric cardia intestinal and Lauren diffuse histological subtypes with a decrease in the incidence of non-cardia intestinal subtypes [3, 4]. There has been a constant rise in the incidence of signet ring cell cancers (SRCC) as well [4–8].

According to the WHO classification, signet ring cell (SRC) histology is a weakly cohesive type of cancer, where more than 50% tumor contains extracellular cytoplasmic mucin and a crescent-shaped nucleus [9, 10]. The recent changes in the histopathological classification have increased the reporting and incidence of SRC histology [11]. SRCC of stomach and GEJ has distinct characteristics such as younger age at presentation, female predominance, advanced stage, lymphatic spread, peritoneal metastasis, and rapid progression [7, 12, 13]. Although there are numerous studies on the prognostic value of SRCC, they are markedly inconsistent in their results, making it impossible to draw any meaningful conclusions. While some studies have indicated better prognosis and an improved survival rate, others have claimed SRCC as a marker for weak prognosis [7, 12, 14–16]. Recent research, however, has shown that SRCC is positively associated with survival outcomes in early-stage gastric/GEJ cancer, while they exhibit worse prognosis in advanced stage compared to non-SRCC [8, 13, 17]. Another area of controversy is the role of neoadjuvant therapy, i.e., neoadjuvant chemotherapy or neoadjuvant chemoradiotherapy in SRCC of stomach and GEJ adenocarcinoma [14, 16, 18–23]. This controversial sensitivity of SRCC towards conventional perioperative chemotherapy regimens and neoadjuvant chemoradiotherapy highlights a significantly specific sensitivity profile to SRCC. Thus, due to lack of significant evidence, SRCC treatment algorithm is still debatable. It is still unclear whether advanced SRCC patients should be primarily resected or considered for multimodal treatment protocols.

The multistage carcinogenesis involving genetic and epigenetic aberrations may have a major role in the increasing incidence of SRCC among gastric and GEJ cancers. It could also be interlinked with stage dependent prognosis, poor survival rate, and reduced chemo/radiotherapy sensitivity of SRCC. However, very few studies have examined SRCC at biomolecular levels [24–26]. Hence, profound knowledge of the SRCC disease course, connected to molecular modifications, is vital in developing personalized approaches for treatment planning and thereby improving the survival rate of the patients.

## Methodology

**Fig. 1:**
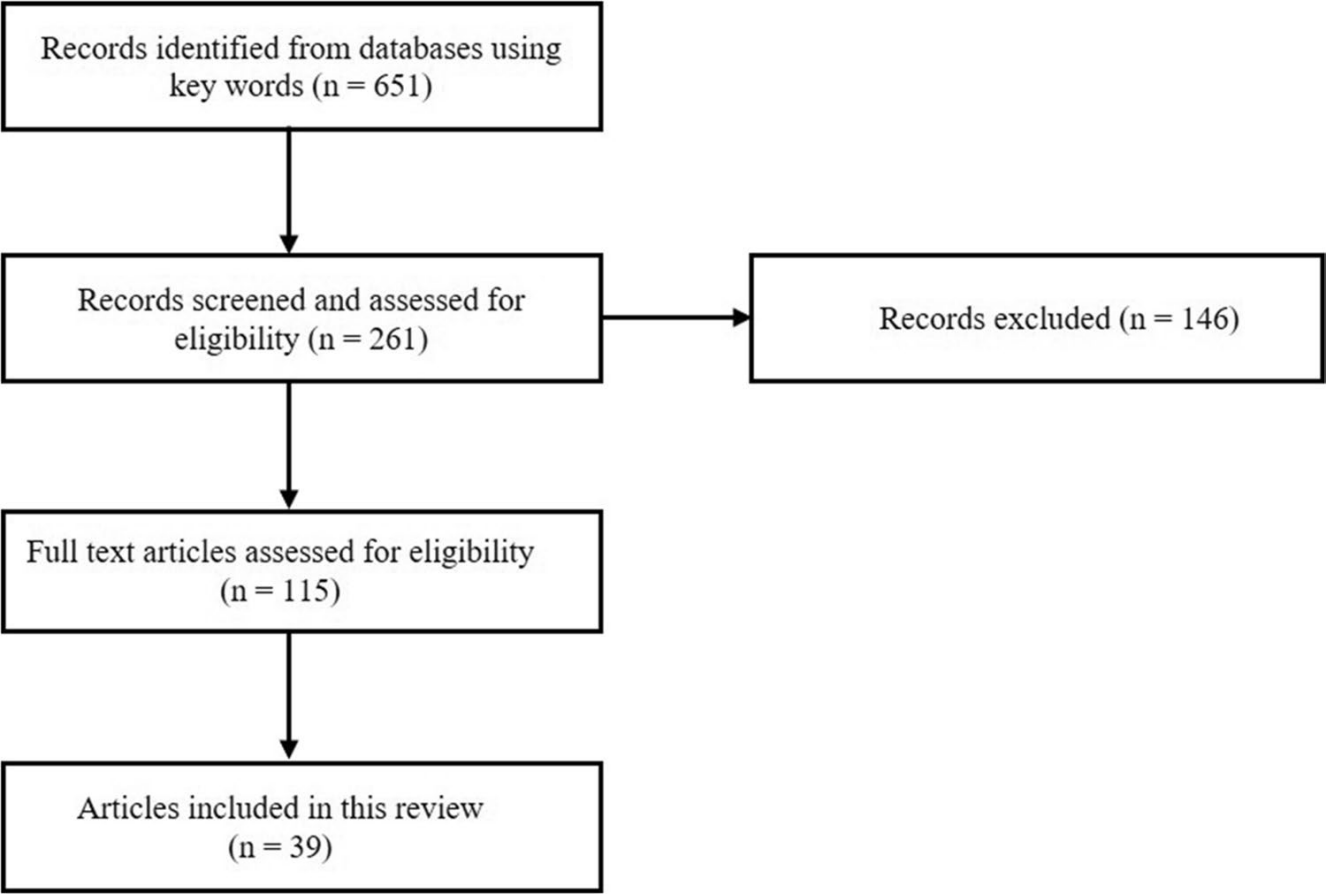
Flow chart of the study selection

A systematic search was carried out in PubMed. Studies available in English related to SRCC of stomach and gastro-esophageal junction were identified and evaluated. Keywords used were “signet ring cell cancer of stomach and gastro-esophageal junction, molecular alterations, genes, biomarkers, and stage stratified treatment”. The results of literature review were descriptively reported in this study (Fig. 1).

## Molecular alterations

### Pathways involved in SRCC of stomach and GEJ carcinogenesis

**Fig. 2:**
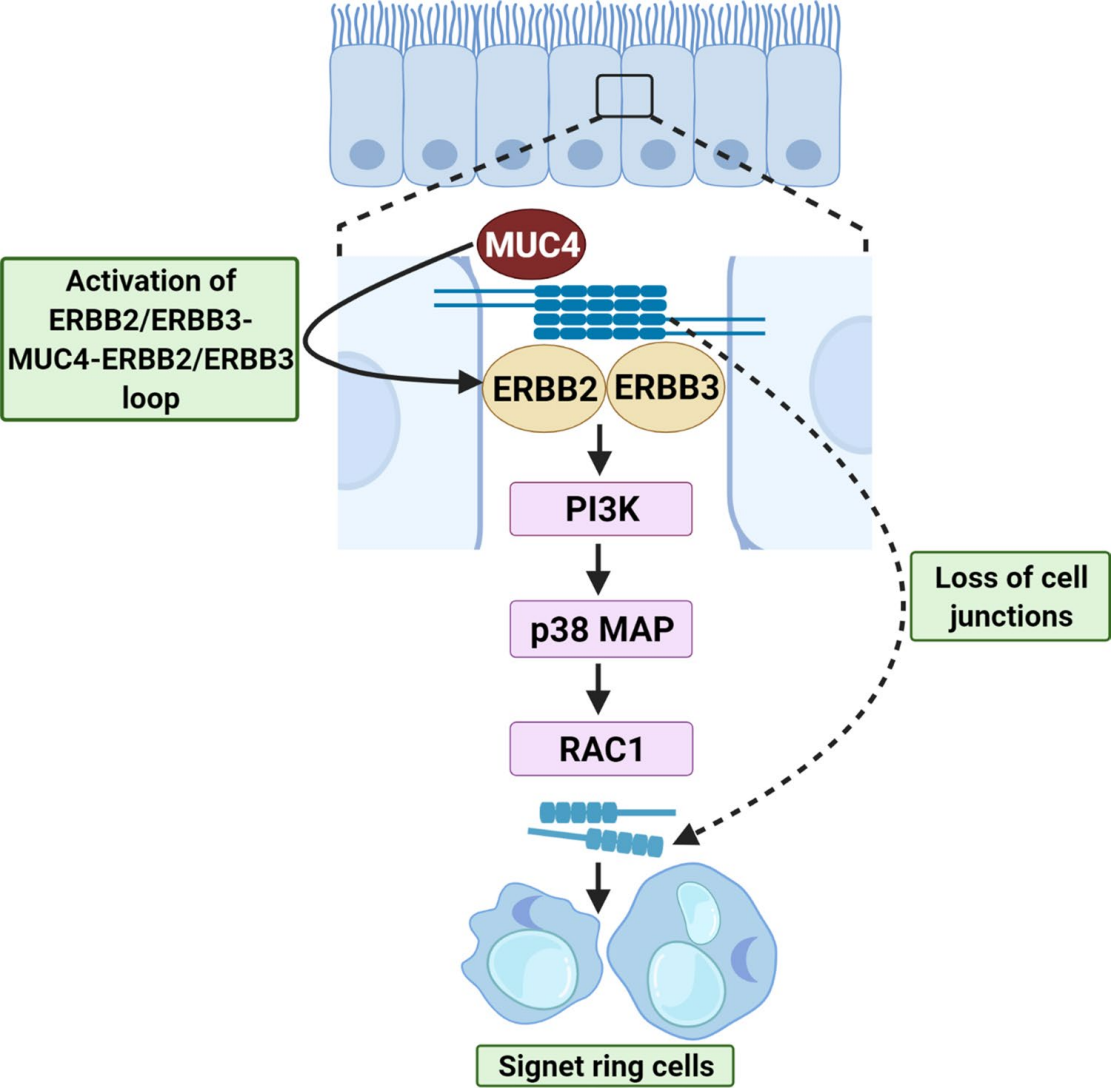
Mechanism involved in SRCC of stomach and GEJ pathogenesis. MUC4, mucin 4; ERBB2, human epidermal growth factor-like receptor 2; ERBB3, human epidermal growth factor-like receptor 3; PI3K, phosphatidylinositol 3-kinase; p38 MAP, p38 mitogen-activated protein kinases; RAC1, Rac family small GTPase 1. Created with BioRender.com

The damage of cell–cell adhesion molecules and accumulation of mucin in large vacuoles are two major pathologic characteristics of SRCC. The loss of function of E-cadherin gene (CDH1) is often considered as the key cause of SRCC and mutations in the E‐cadherin gene occurs during initial phases of SRCC [27, 28]. In highly differentiated adenocarcinoma, activation of human epidermal growth factor-like receptors 2 and 3 complexes (ERBB2 and ERBB3) followed by phosphatidylinositol 3-kinase (PI3K) and Rac family small GTPase 1 (RAC1) activation leads to disruption of adherence junctions and secretion of mucins. One of the mucins, MUC4, has been described to surge activation of the ERBB2/ERBB3 complex and, this continuous activation of ERBB2/ERBB3-MUC4 loop, leads to loss of tight junctions and cell–cell interactions, which further leads to formation of SRC [11, 29]. Therefore, large vacuoles formed due to accumulation of mucins possibly play a title role in carcinogenesis (Fig. 2).

### Genetic factors involved in SRCC of stomach and GEJ

The influence of genetic factors in the development and prognosis of SRCC is now well recognized. The influence of these factors is well established to be due to the involvement of E-cadherin gene mutation in SRCC pathogenesis. The loss of E-cadherin was a recurrent event in SRCC of several organs, and this was more prominent in SRCC than in non-SRCC of similar tumors [30]. The reduced E-cadherin in primary SRCC supports its role during epithelial-mesenchymal transition (EMT) in tumor development and metastasis; however, its re-expression benefits tumor cells to form solid metastatic deposits [31]. Moreover, heterozygous germline CDH1 mutations intensify the risk of developing diffuse gastric cancer (DGC) and lobular breast cancer (LBC). In addition, individuals with CDH1 mutation are approximately at 70% risk of developing DGC by 80 years of age [32].

In a study conducted by Tamura G et al., 57% (8/14) of SRCC of stomach samples exhibited E-cadherin promoter hypermethylation and was significantly associated with reduced expression of E-cadherin [33]. Epigenetic deactivation of E-cadherin via promoter hypermethylation has been regarded as a fundamental step in the progression of undifferentiated tumors [34]. In metastatic gastric SRCC, programmed death ligand 1 (PDL1) expression was associated with poor prognosis [35]. Moreover, in advanced gastric cancers with SRCC, CD3+ T cell infiltration was more in PD-L1 positive tumors, which could be a lead for further investigation of immunotherapy markers in SRCC of stomach [36]. Additionally, epithelial membrane antigen (EMA) and caudal type homeobox 2 (CDX-2) genes were found under expressed in primary gastric SRCC compared with normal gastric epithelium [37]. A study by Yue G et al. identified over expression of transcriptional coactivator with PDZ-binding motif (TAZ) in gastric SRCC suggesting TAZ as a potential drug target for SRCC treatment [38].

Several scientific literatures reveal that many genes, in addition to the conventional genes are involved in SRCC. A whole-genome study conducted in 32 pairs of gastric SRCC patient samples identified six significantly mutated genes: TP53 (25%), CDH1 (15.6%), PIK3CA (12.5%), ERBB2 (6.3%), LCE1F (6.3%), and OR8J1 (6.3%) [39]. High-content signet ring cell cancer (HSRCC), which contain more than 80% of signet ring cells, consistently showed high frequency of TP53 alteration, multiple oncogene amplification, and cell adhesion-related gene mutations. Nevertheless, recurrent amplification in MYC and BCAS1 genes, along with low mutation rate in ARID1A and RHOA, recommends genetic differences between HSRCC and other subcategories of gastric cancer [39]. High frequency of gastric cancer specific fusions (i.e., CLDN18-ARHGAP26/6) has been detected in the HSRCC specimens. The occurrence of CLDN18-ARHGAP26/6 fusion was associated with SRCC content, age at diagnosis, female/male ratio, and TNM stage. Moreover, patients with CLDN18-ARHGAP26/6 fusion had poor survival outcomes and received no improvement from oxaliplatin-/fluoropyrimidine-based chemotherapy [39]. Zhao ZT et al., through transcriptome analysis, identified upregulation of MAGEA (melanoma antigen gene A) family members including MAGEA2, MAGEA3, MAGEA4, and MAGEA6 and downregulation of REG1B. As MAGEA family is categorized under cancer testis antigen, it is considered as an attractive target for adoptive immunotherapy [40].

**Fig. 3:**
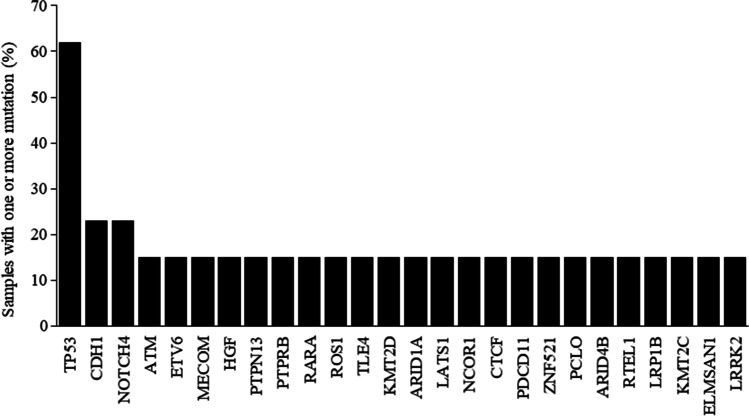
Top mutated cancer genes in gastric SRCC retrieved from cBioPortal-TCGA, PanCancer Atlas data. (http://www.cbioportal.org/). SRCC, signet ring cell carcinoma; TCGA, the cancer genome atlas; TP53, tumor protein p53; NOTCH4, notch receptor 4; ATM, ataxia telangiectasia mutated; ETV6, ETS variant transcription factor 6; MECOM, MDS1 and EVI1 complex locus; HGF, hepatocyte growth factor; PTPN13, protein tyrosine phosphatase non-receptor type 13; PTPRB, protein tyrosine phosphatase receptor type B; RARA, retinoic acid receptor alpha; ROS1, ROS proto-oncogene 1; TLE4, transducin-like enhancer protein 4; KMT2D, lysine methyltransferase 2D; ARID1A, AT-rich interaction domain 1A; LATS1, large tumor suppressor kinase 1; NCOR1, nuclear receptor corepressor 1; CTCF, CCCTC-binding factor; PDCD11, programmed cell death protein 11; ZNF521, zinc finger protein 521; PCLO, piccolo presynaptic cytomatrix protein; ARID4B, AT-rich interaction domain 4B; RTEL1, regulator of telomere elongation helicase 1; LRP1B, LDL receptor-related protein 1B; KMT2C, lysine methyltransferase 2C; ELMSAN1, mitotic deacetylase-associated SANT domain protein; LRRK2, leucine-rich repeat kinase 2

The Cancer Genome Atlas (TCGA) PanCancer atlas evaluated 440 stomach adenocarcinomas, out of which 13 samples were SRCC [41]. The top mutated cancer genes in SRCC, retrieved from cBioPortal for cancer genomics database, are shown in Fig. 3 [42, 43]. Besides, seven cancer genes including CTNNA1, ERBB2, MECOM, NFE2L2, PRKCI, TRAF3, and ARHGAP26 were found to be fusion genes. CLTC, ERBB2, FAT1, IRF2, KDR, KIT, LIFR, and MDM2 were some among the cancer genes which displayed copy number alterations (CNA) in gastric SRCC samples [42, 43].

### Biomarkers

Level of microRNA expression has been considered as a potential biomarker for cancer diagnosis and prognosis. Dysregulated gene expression by miRNA in the post-translational level is highly related to pathophysiology of cancer. Although numerous studies have revealed the role of miRNA in gastric cancer tumorigenesis, very few studies have explored the role of miRNA in gastric SRCC. In a study conducted by Chen J et al., high incidence of invasive metastases and chemoresistance of gastric SRCC were associated with downregulation of hsa-miR-665 and hsa-miR‑95 [44]. miRNA microarray analysis by Li FQ et al. identified thirteen dysregulated miRNAs in SRCC compared with tubular adenocarcinoma [45]. A recent study by Saito R et al. reported overexpression of miR-99a-5p predominantly in the primary stage SRCC which resulted in inhibition of cancer cell proliferation. Moreover, high miR-99a-5p expression correlated with less aggressive clinicopathological characteristics. Thus, miR-99a-5p can be considered as a diagnostic and prognostic biomarker in patients with early stage SRCC [46].

An endo-β-D-glucuronidase, heparanase (HPA), was found to be overexpressed in gastric SRCC, compared to non-SRCC. Pro-metastatic and pro-angiogenic properties of HPA make it an ideal tumor biomarker in gastric SRCC [47]. According to a study by Chen TH et al., advanced SRCCs could be graded into prognostically unique subcategories based on N-acetylgalactosaminyltransferase 14 (GALNT14) genotyping. Patients with GALNT14-rs9679162 “TT” genotype exhibited weak postoperative prognosis in advanced gastric SRCC. The study also confirmed that GALNT14-rs9679162 “TT” genotype could aid as a significant prognostic biomarker in gastric SRCC subgroups with aggressive phenotypes [48].

Khan SA et al. found that the combination of altered histone modifications, H4K16ac, and H4K20me3 along with H3S10ph serve as molecular prognostic markers for gastric cancer and concluded that increased H3S10ph in GC might assist in defining true negative surgical resection margin [49]. Moreover, analysis of differential activity and expression levels of class 1 histone deacetylases (HDACs) in patient samples and TCGA database suggested a solid association among global histone hypo-acetylation with increased HDAC activity in both gastric cancer tissue samples and cell lines [50].

A recent study, investigating the association between DGC histologic subtypes and expression of Wnt target genes showed that SRCC morphology was regulated by Wnt and R-spondin expression and highlighted how genetic mutations influence DGC phenotypes [51]. Moreover, SRCC patients with KRAS mutation were identified with lower overall survival rate [52]. In gastric SRCC, estrogen receptor beta (ERβ) inhibited the cell proliferation and invasiveness via mTOR–Arpc1b/EVL signaling pathway, and thus, ERβ might be considered as a potential target for SRCC treatment [53]. These studies confirm that a thorough understanding of the molecular alterations linked to gastric SRCC is required to guide surgical and medicinal treatment approaches [24].

## Early gastric SRCC

Early gastric cancer (EGC) is characterized by involvement up to submucosal layer (cT1) of stomach regardless of lymph node metastasis. Endoscopic resection including endoscopic mucosal resection (EMR)/endoscopic submucosal dissection (ESD) or radical surgery is the recommended lines of treatment in EGC. In EGC, SRCC is defined as more than 50% cancer cells in the mucosa [13]. Definitive surgery is the recommended line of treatment in SRCC, and endoscopic treatment is selectively considered. When patients are positive for CDH1 mutations, radical total gastrectomy with extensive lymphadenectomy is suggested [24].

As per the extended criteria by Gotoda T et al., ESD can be performed in EGC with undifferentiated histology including SRCC [4, 54]. However, patients with EGC having ulcerated tumor limited to mucosa, lesions larger than 3 cm, undifferentiated histology, and early lymphatic invasion have increased risk of lymph node metastasis [11]. Chung JW et al. reported 1.15% (3/261 patients) of lymph node metastasis in a series of 1721 patients with tumors of undifferentiated histology, less than 2 cm with no ulceration [55]. Ha TK et al. stated no lymph node metastasis in 77 patients with EGC with SRC confined to the mucosa and less than 2 cm in size and with no lymphatic involvement [56]. Another concern for endoscopic surgery is the lateral resection margin. The infiltrative type of SRCC has the tendency for subepithelial spread with normal appearing mucosa, hence the importance of wider lateral margins [57, 58]. Due to these reasons, various endoscopic guidelines have been established. Ahn JY et al. demonstrated oncological safety after curative endoscopic resection following extended criteria [59]. Japan Gastric Cancer Association (JGCA) treatment guidelines also recommends extended criteria for undifferentiated histology; however, ambiguity still persists regarding oncological safety of therapeutic endoscopic approach [60]. In the west, EGC has higher reported rate of lymph node metastasis than those reported in eastern studies [4, 61]. Recent studies have also reported higher rate of lymph node spread in SRCC [62, 63]. This could be largely due to difference in histopathological classification and difference in cancer biology [4]. Hence, endoscopic resection is not routinely recommended in the west for diffuse histology [64]. A standardized histopathological classification for both the East and the Western group and an in-depth research on molecular alterations would give a definitive answer in the future. Until then endoscopic resection is limited to highly selected patients with early gastric SRCC.

The prognosis of early gastric SRCC has been reported to be equivalent or better than non-SRC histology in several studies [11, 13, 24]. The survival rate is also better than non-SRCC [11]. A retrospective study by Ha TK et al. including 1520 EGC patients reported better survival rate in SRCC than non-SRC histology [56]. Kao YC et al. also demonstrated better 5-year overall and disease-free survival in early gastric SRCC [13]. The better survival in SRCC is most likely due to presentation at younger age, tumor limited to mucosa, and lesser involvement of lymph nodes [11]. Therefore, SRC histology may not be an independent predictor for overall survival in EGC [13]. Thus, radical surgery with extended lymphadenectomy in patients with high risk for lymph node metastasis and reserving endoscopic resection in highly selected patients will give better oncological outcomes in early gastric SRCC.

## Advanced gastric/GEJ SRCC

Advanced gastric or GEJ cancer is defined by involvement of at least muscular layer (cT2, T3, T4). In advanced gastric cancer, SRCC is defined as more than 50% cancer cells in the mucosa irrespective of deep invasive component [13]. Kao YC et al. reported that advanced SRCC patients were younger, greater female/male ratio, larger tumor size, involvement of body or distal stomach, poorly differentiated histology, more advanced Borrmann type, more scirrhous-type stromal reaction, more lymphovascular invasion, greater tumor depth of invasion, and more lymph node metastasis [13]. Due to high risk of distant lymph node metastasis and higher tendency for peritoneal dissemination, treatment of advanced SRCC is quite challenging. Curative gastrectomy with radical lymph node dissection is the recommended line of treatment in advanced SRCC. However, role of perioperative chemotherapy, neoadjuvant chemoradiotherapy, cytoreductive surgery (CRS), and hyperthermic intraperitoneal chemotherapy (HIPEC) in advanced SRCC have been explored in many studies, though not well established due to debatable responses to these therapeutic modalities.

The surgery for SRCC includes subtotal or total gastrectomy with wide margins and D2 lymphadenectomy. The debate continues on optimum resection margin due to subepithelial spread and the role of extended lymphadenectomy due to higher risk for regional and distant lymph node metastasis. Piessen G et al. reported higher rate of positive resection margin even after an extensive surgery [65]. Therefore, wider resection margin should be aimed for SRCC than for any other histology [4]. In the era of minimally invasive surgery, laparoscopic distal gastrectomy is non inferior to open surgery [66]. The CLASS-01 randomized clinical trial which included 15% of SRCC in laparoscopic arm showed no difference in 3-year disease free survival between laparoscopic and open distal gastrectomy with D2 lymphadenectomy [66]. On the other hand, a study by Kelly KJ et al. reported that 75% of margin positivity was associated with SRC histology in the laparoscopic group [67]. Therefore, laparoscopic approach should be considered only in carefully selected patients with SRCC. Though D2 lymphadenectomy is the standard of care for advanced gastric cancer, role of D3 lymph node dissection has been explored in many studies without any oncological benefit [68]. The Italian Research Group for Gastric Cancer (GIRCG) in a retrospective study reported lower recurrence rate in advanced gastric cancer with diffuse histology following D3 lymphadenectomy [69]. Due to the lymph tropism and higher tendency for metastasis to D3 lymph nodes in diffuse histology [4, 70], the role of extended lymphadenectomy needs to be established in future studies.

Perioperative chemotherapy is the recommended line of treatment for locally advanced gastric cancer [71]. However, due to debatable chemosensitivity in SRCC, controversy still exists on whether to offer neoadjuvant chemotherapy or to go ahead with upfront surgery. A multicentric study by FREGAT working group (FRENCH) reported no survival benefit with perioperative fluorouracil-platinum doublet or triplet chemotherapy in gastric SRCC [14]. Lack of cytostatic and cytotoxic effects of chemotherapy on SRCC and delay in definitive surgery during neoadjuvant period may lead to tumor progression and result in poorer outcomes [14, 55]. However, Heger U et al. reported improved oncological outcomes even with less frequent clinical and pathological response to neoadjuvant chemotherapy [72]. The FLOT4 trial, comparing perioperative FLOT versus perioperative ECF/ECX in locally advanced resectable gastric or gastro-esophageal junction adenocarcinoma that included 28% of SRCC, showed improved survival outcomes with FLOT regimen [73]. Heger U et al. in a recent study evaluated neoadjuvant chemotherapy (both FLOT and EOX chemotherapy) versus upfront surgery in locally advanced SRC esophagogastric adenocarcinoma and demonstrated survival advantage with neoadjuvant strategy [18]. On the other hand, a small retrospective study by Li Y et al. reported no survival benefit and recommended upfront surgery in SRCC [19]. In the ongoing PRODIGE 19–FFCD1103–ADCI002 trial evaluating the strategy of upfront surgery followed by adjuvant chemotherapy versus perioperative chemotherapy, the authors hypothesize that upfront surgery will have oncological benefit in resectable gastric SRCC [74]. A small data suggests the efficacy of taxane-based chemotherapy in SRCC [11]. With insufficient data currently available, advanced SRCC may be selectively treated with perioperative FLOT chemotherapy or upfront surgery. The effect of FLOT chemotherapy versus upfront surgery in SRCC needs to be explored in future prospective studies.

The treatment of locally advanced GEJ is another area of controversy. Neoadjuvant chemotherapy [73] and neoadjuvant chemoradiotherapy as suggested by CROSS trial [75] are the recommended treatment options. A small study by FREGAT working group (FRENCH) suggested good response and better survival outcomes following neoadjuvant chemoradiotherapy compared to upfront surgery in SRCC of GEJ [21]. A recently published retrospective study by van Hootegem SJM et al. also reported greater tumor downstaging and better disease-free survival with neoadjuvant chemoradiotherapy compared to neoadjuvant chemotherapy in SRCC GEJ [16]. Further prospective studies would be required to establish neoadjuvant chemoradiotherapy followed by surgery as an optimal treatment strategy for SRCC of GEJ.

Peritoneal metastasis in SRCC could be synchronous or metachronous [4]. High rate of unsuspected peritoneal dissemination at the initial diagnosis is well known with SRCC [65, 76]. Nearly half of the patients with SRCC have peritoneal recurrence even after receiving standard surgery [65, 77–79]. The predictive factors for peritoneal metastasis are the presence of linitis plastica, invasion of the peritoneal serosa or beyond, and associated lymph node metastasis [78]. Due to these reasons, the role of both prophylactic and therapeutic CRS and HIPEC needs to be explored in SRCC. In patients with limited peritoneal metastasis, CRS and HIPEC might play a role due to better response rate [24]. In the recently published CYTO-CHIP study, 60% of gastric SRCC showed improved OS and recurrence-free survival, without additional morbidity or mortality in selected patients with limited peritoneal metastasis and low peritoneal carcinomatosis index (PCI) [80]. Another multicentric study of Spanish Group of Peritoneal Oncologic Surgery (GECOP) reported improved survival outcomes in selected patients (PCI < 7) following CRS and HIPEC [81]. The perioperative chemotherapy using the FLOT protocol followed by CRS + HIPEC seems to be more effective in selected patients [82]. Thus, therapeutic CRS and HIPEC can be considered in highly selected SRCC patients with low PCI following perioperative chemotherapy. A systematic review on prophylactic HIPEC for gastric cancer by Brenkman HJF et al. including three randomized controlled trials and eight non-randomized comparative studies reported better oncological outcomes [83]. However, these results need to be replicated with prospective randomized controlled trials. The results of a prospective, open, randomized multicentric phase III clinical study (GASTRICHIP) evaluating the outcomes of prophylactic HIPEC in gastric cancer involving the serosa and/or lymph node involvement and/or with positive cytology at peritoneal washing treated with perioperative systemic chemotherapy and D1–D2 curative gastrectomy is highly awaited [84]. Until then prophylactic HIPEC can be considered only in clinical trial settings.

The prognosis of advanced SRCC is controversial. Few reports suggest poor prognosis, while other studies illustrate that SRC histology is not an independent predictor after adjustment for stage [11]. Kao YC et al. suggest that SRC histology is an independent predictor for overall survival as well as a poor prognostic factor in advanced SRCC after curative surgery [13]. A meta-analysis by Nie RC et al. indicates that advanced SRCC is associated with poor prognosis [17]. Voron T et al. reported worse prognosis, different prognostic factors, and poor response to perioperative chemotherapy and concluded that SRCC should be considered as a specific entity [85]. A study by Taghavi S et al. reported that SRC histology did not portend a worse prognosis when adjusted for stage in patients of the USA [7]. A stage-stratified analysis of SRCC versus intestinal-type tumors by Bamboat ZM et al. suggested that long-term outcomes in SRCC is affected by the extent of disease rather than the mere presence of SRC histology [8]. The authors hypothesize that driver mutations responsible for metastasis may occur as the stage advances [8]. The poor prognosis in advanced stage is mostly due to multiple factors like aggressive SRC phenotype, high risk for lymph node and peritoneal metastasis, involvement of adjacent organs, differential response to neoadjuvant treatment, and lower R0 resection rate. Future studies on genetic, molecular, and tumor microenvironment analysis would give a definitive answer [8, 47].

## Recommendations for treatment of SRCC of stomach and GEJ

- Endoscopic resection with wider margin is recommended only for highly selected early gastric SRCC, confined to the mucosa without ulceration, less than 2 cm in size and with no lymphatic involvement.
- Radical surgery with wide margin and D2 lymphadenectomy is recommended line of treatment for early gastric SRCC with high risk for lymph node metastasis.
- Laparoscopic surgery should be considered only in carefully selected patients with early gastric SRCC.
- Advanced SRCC is selectively treated with perioperative FLOT chemotherapy or upfront surgery followed by adjuvant chemotherapy.
- Curative gastrectomy with wide margin and D2 lymph node dissection is the recommended line of treatment in advanced SRCC.
- In selected patients with limited peritoneal metastasis and low PCI, therapeutic CRS and HIPEC can be considered following perioperative chemotherapy using the FLOT protocol.
- The recommended treatment options for locally advanced GEJ are neoadjuvant chemotherapy or neoadjuvant chemoradiotherapy followed by definitive surgery.

## Future directions

Standardization of terminology and histopathological classification across the globe would be the need of the hour. In-depth research on SRCC based on molecular alterations and gene expressions is important to improve the outcomes. The identification of molecular mechanisms would provide information on tumorigenesis and tumor progression and subsequently could be used to develop therapeutic agents [86]. The development of diagnostic biomarkers would help in prognostication and identification of potential therapeutic targets. The characterization of comprehensive genomic features through transcriptome sequencing and multiple driver mutations needs to be learned [40]. The prognostic stratification of SRCC can be done by clinicopathological factors and GALNT14 genotype as suggested by Chen TH et al. [48]. Future studies may need to focus on chemosensitivity profile in SRCC. The sensitivity of taxane-based chemotherapy to SRCC as demonstrated in subgroup analysis of FLOT trial needs to be studied prospectively. With poor response to chemotherapy, future studies need to explore targeted molecular therapy aiming the EMT pathway. The genes and pathways involved in the pathogenesis and progress of SRCC constitute important targets for chemical inhibitors, which can improve the prognosis of advanced SRCC patients.

The slow advancement of gastric SRCC in clinical practice might be due to the absence of a systematic molecular overview of this disease. This review urges the need for combining the knowledge on molecular and pathological involvement, to address the inconsistency in SRCC diagnosis and management.

With respect to surgery, the role of extended lymphadenectomy and prophylactic and therapeutic CRS and HIPEC needs further level 1 evidence. The careful selection of patients for perioperative chemotherapy versus upfront surgery can be directed based on prognostic biomarkers.

## Conclusion

Multistage carcinogenesis involving genetic and epigenetic aberrations in SRCC is interlinked with stage-dependent prognosis. Specific therapeutic strategy and personalized multimodal treatment should be followed based on the tumor characteristics of SRCC. Endoscopic resection, radical surgery, and perioperative chemotherapy should be offered in carefully selected patients based on stage and prognostic stratification. Future studies in genetic and molecular analysis, histopathological classification, and options of multimodality treatment will improve the prognosis and oncological outcomes in SRCC of gastric and GEJ.

## Data Availability

Not applicable
